# Impact of hearing loss on the performance of auditory processing measured by questionnaires in Korean adolescents

**DOI:** 10.1038/s41598-020-67033-2

**Published:** 2020-06-22

**Authors:** Joong Ho Ahn, Seung-Ha Oh, Hyunsook Jang, Jung-Bok Lee, Jong Woo Chung

**Affiliations:** 1 Department of Otorhinolaryngology-Head and Neck Surgery, Asan Medical Center, University of Ulsan College of Medicine, Seoul, Korea; 2 Department of Otorhinolaryngology-Head and Neck Surgery, Seoul National University Hospital, Seoul National University College of Medicine, Seoul, Korea; 3 Division of Speech Pathology and Audiology, Research Institute of Audiology and Speech Pathology, College of Natural Sciences, Hallym University, Chuncheon, Korea; 4 Department of Biostatistics, Asan Medical Center, University of Ulsan College of Medicine, Seoul, Korea

**Keywords:** Health care, Risk factors

## Abstract

Increasing use of personal listening devices has been accompanied by increase in the prevalence of hearing loss (HL) among youth in Korea, as in other countries. Auditory processing disorder (APD) is one of the main factors affecting academic achievement at school. This study aimed to investigate the prevalence of HL in students attending general middle- and high schools and compare the findings with the APD survey results. From June 1 to December 31, 2016, Korean adolescents (n = 2,791) in the first years of middle- and high school underwent audiometric testing and otologic examination and completed questionnaires on APD. The survey was sponsored by the Korean Society of Otolaryngology-Head and Neck Surgery and the Korean Otology Society. The prevalence of speech-frequency hearing loss (SFHL) and high-frequency hearing loss (HFHL) in the poorer ear was 11.6% and 10.3%, respectively, among Korean adolescents. We analysed data from the Scale of Auditory Behaviors, Fisher’s Auditory Problems Checklist, and KNISE-Auditory Behavioral Checklist and compared these with the results of hearing tests. We observed positive correlations among the APD questionnaire results and mean hearing levels. This study suggested that hearing loss, especially bilateral high-frequency hearing loss, may affect central auditory processing.

## Introduction

With the increasing use of personal listening devices, the prevalence of hearing loss (HL) among the current generation of adolescents has been predictably higher than that among the past generations of adolescents. A study from the USA reported that the prevalence of HL among US adolescents increased from 14.9% in 1988–1994 to 19.5% in 2005–2006. In addition, chronic noise exposure from using mobile devices may increase high-frequency hearing loss (HFHL)^1^. Two nationwide studies have been conducted on adolescents in South Korea: Hong *et al*. reported unilateral and bilateral HFHLs > 20 dB in 5.0% and 1.9%, respectively, of 1,658 adolescents aged 13–18 years in South Korea^2^ and Rhee *et al*. reported that approximately 17% of Korean adolescents exhibited at least slight HL^3^.

Understanding external auditory speech normally and naturally requires at least two stages: auditory processing of the signal and language-based processing of that information^4^. Because of masking effects from background noise or a hearing problem, the acoustic speech signal is easily degraded, making it difficult to understand. To overcome this difficulty and facilitate speech perception, one strategically adopts some auditory and language-based compensatory mechanisms^5^. Such language-based mechanisms include semantic and lexical expectations about words presented in context to facilitate speech perception in a noisy environment^4,6,7^. However, unlike adults who have normal language fluency, children in the process of learning a language lack the capacity to “fill in the blanks” for inaudible sounds. Moreover, it is often challenging for children to understand speech under various listening conditions, such as a noisy or a reverberant environment^8^.

Auditory processing disorders (APDs) are defined as speech perception deficits that exist despite normal peripheral hearing^9^, and approximately 2–5% of school-aged children are reported to be affected by hearing problems^10^. In addition, a 2017 New Zealand study of youth offenders aged 14–17 years reported that 27% suffered from APD and 64% showed language impairment compared to 18% and 10% of adolescents with normal hearing, respectively^11^. Children diagnosed with APD have listening difficulties, and many of those who present at audiology or other paediatric clinics have ‘normal’ audiograms. Most of these children have other complications such as speech/language or attention impairments and various learning disorders^12^.

Although the central hearing difficulty seems to be a single problem, it may be a cause of language disorder, reading disorder, learning disorder, attention deficit hyperactivity disorder, and other problems^13,14^. Therefore, it must be diagnosed as early as possible. Several test batteries are available to diagnose APDs in patients with normal hearing^15,16^. However, several studies have shown that in patients with cochlear HL of up to approximately 45–50 dB, speech perception is mainly influenced by audibility^17,18^. In patients with more severe HL, poor discrimination of suprathreshold stimuli plays an important role in addition to the loss of audibility.

However, APD was found in children with normal pure tone average or mild high-frequency hearing loss^12,19^. Therefore, having normal pure tone average does not mean that speech perception is normal.

The purpose of this study was to analyse the relationship between different degree of HL and results of screening surveys of the central auditory processing ability of middle- and high-school students in South Korea.

## Methods

### Study design and participants

We performed a cross-sectional, complex sampling survey of first-year middle- and high-school students, except those attending special schools for disabled children. The sampling frame was the 2015 Yearbook of Educational Statistics prepared by the Korean Educational Statistics Services^20^.

We sought to enrol 3,013 students from 124 middle schools and 124 high schools based on the prevalence of HL in Korean adolescents, the estimated association between noise and HL, and an expected 80% response rate^21,22^. Details of the study design and research methods are presented in another study^2^.

The survey was performed from June 1 to December 31, 2016. After providing written information about the study to students and parents, we selected and evaluated only those children who themselves and whose parents provided written informed consent. This study was approved by the Institutional Review Board of Seoul National University Hospital (No. 1604-086-755). All methods/experiments were performed in accordance with relevant guidelines and regulations (Declaration of Helsinki).

### Data collection and measurements

#### Audiological evaluation

The methods of audiological evaluation are superbly detailed in the previous study^2^. Briefly, four experienced audiologists measured the hearing thresholds of adolescents using an AD229b diagnostic audiometer (Interacoustics, Assens, Denmark) in a soundproof booth inside a mobile vehicle. Prior to audiometry, otological examinations were performed to rule out factors affecting hearing, such as external ear anomalies, ear wax, retraction of the tympanic membrane, cholesteatoma, and middle ear effusion.

The tested frequencies were 0.5, 1, 2, 3, 4, 6, and 8 kHz. We started at 30 dB, adjusting with a 10 dB decrease or a 5 dB increase according to the participant’s response, until we decided that his or her frequency-specific hearing threshold was reached. If the hearing threshold was over 25 dB at any frequency, we measured the bone conduction threshold to determine whether conductive hearing loss was present.

We defined HL as a hearing threshold ≥15 dB. The severity of HL was based on the poorer threshold for unilateral HL and better threshold for bilateral HL, as described previously^1^. Speech-frequency hearing loss (SFHL) was the average of the HLs at 0.5, 1, and 2 kHz, while HFHL was the average of the HLs at 3, 4, 6, and 8 kHz.

#### Questionnaires

The students were asked to complete separate questionnaires to evaluate their everyday difficulties in areas such as listening, learning, and communication.

The KNISE-Auditory Behavioural Checklist (KNISE-ABC) is used for screening children with APDs and other related disorders to provide information necessary for establishing and implementing management and educational plans^22–24^. The KNISE-ABC has 36 items rated on a 5-point scale and includes seven test areas, namely, listening, listening in background noise, communication, learning, auditory memory, auditory attention, and related behaviour.

The Scale of Auditory Behaviours (SAB) questionnaire, chosen for this study, has a version that may be used by parents or professionals^25^. The questionnaire is easily administered because it has a small number (12 items rated on a 5-point scale) of questions and answer options that are easily understood. This questionnaire was developed to assess everyday behaviours associated with audition, listening and academic skills, attention, memory, and organisation.

Fisher’s Auditory Problems Checklist (FAPC) was developed by Fisher L^26^ and comprises a list of 25 items rated on a 2-point scale. To collect information about the perceived auditory processing problems, this checklist includes questions on acuity, attention, attention span, figure ground, discrimination, short-term memory, long-term memory, sequential memory, comprehension, speech and language problems, auditory-visual integration, motivation, and performance.

#### Statistics

All data in the study are summarised as means and standard deviations for continuous variables and as frequencies and percentages for categorical variables. Comparisons of general characteristics between HL and no HL were conducted using the chi-squared test or Fisher’s exact test. Prevalence rates of SFHL and HFHL were estimated with 95% confidence intervals. To evaluate the association between the degree of HL and mean scores of KNISE-ABC, SAB, and FAPC, we adopted Wilcoxon’s rank sum test. All statistical analyses were performed using SAS (ver. 9.4; SAS Institute, Cary, NC), and statistical significance was defined as a two-sided *p-*value <0.05.

## Results

**Figure 1:**
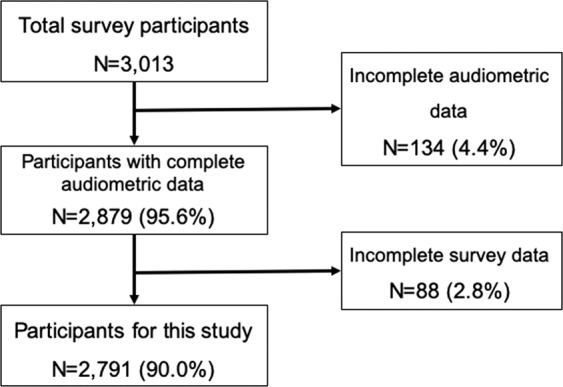
Flowchart of the selection process.

In total, 3,013 students from 109 middle- and high schools agreed to complete the questionnaires. Of these, 134 (4.4%) were excluded because of incomplete audiometric data. Additionally, 88 students were excluded because of incomplete survey tests. Finally, a total of 2,791 (90.0%) students participated in this study (Fig. 1).

Demographic characteristics of these 2,791 students are summarised in Table 1. There were no significant differences between the no-HL and any-HL groups in sex ratio, grade level of middle- vs. high school, annual household income, and family history of hearing loss. Among the 2,791 students, 97.2% showed normal shape of the outer ear and 95.8% had normal otoscopic findings. The prevalence of any-HL was significantly higher in participants with an abnormal outer ear or otoscopic findings (*P* < 0.05).

**Table 1:** Multivariate logistic regression of potential risk factors for HL in Korean adolescents (n = 2,791).

		Total	No HL^†^	Any HL^‡^	P-value^*^
		(n = 2,791)	(n = 2,434)	(n = 357)	
Sex	Male	1,456 (52.2)	1,257 (51.6)	199 (55.7)	*NS*
	Female	1,335 (47.8)	1,177 (48.4)	158 (44.3)	
School	Middle	1,507 (54.0)	1,295 (53.2)	212 (59.4)	*NS*
	High	1,284 (46.0)	1,139 (46.8)	145 (40.6)	
Household income per month, $US	~1,000	76 (3.1)	63 (3.0)	13 (3.6)	*NS*
	1,000~2,000	316 (12.9)	280 (13.1)	36 (10.1)	
	2,000~3,000	481 (19.7)	405 (19.0)	76 (21.3)	
	3,000~4,000	626 (25.6)	546 (25.6)	80 (22.4)	
	4.000~	945 (38.7)	843 (34.6)	102 (28.6)	
	No response	347 (12.4)	297 (12.2)	50 (14.0)	
Family history	Yes	234 (8.4)	208 (8.6)	26 (7.3)	*NS*
	No	2,557 (91.6)	2,226 (91.5)	331 (92.7)	
Outer ear findings	Normal	2,713 (97.2)	2,362 (97.0)	351 (98.3)	<0.05*
	Microtia	6 (0.2)	5 (0.2)	1 (0.3)	
	Preauricular fistula	50 (1.8)	50 (2.1)	0 (0.0)	
	Others	22 (0.8)	17 (0.7)	5 (1.4)	
Otoscopic findings	Normal	2,674 (95.8)	2,352 (96.6)	322 (90.2)	<0.05*
	Perforation	3 (0.1)	2 (0.1)	1 (0.3)	
	Retraction	4 (0.1)	1 (0.0)	3 (0.8)	
	Otitis media	23 (0.8)	9 (0.4)	14 (3.9)	
	Others	87 (3.1)	70 (2.9)	17 (4.8)	

Chi squared test or *Fisher’s exact test

^†^No HL, hearing threshold <15 dB; ^**‡**^Any HL, pure tone average of either ear better at high frequencies [3, 4, 6, and 8 kHz] or speech frequencies [0.5, 1, and 2 kHz] ≥ 15 dB.

HL, hearing loss; NS, not significant.

The prevalence of any-HL (pure tone average of either ear better at high frequencies [3, 4, 6, and 8 kHz] or speech frequencies [0.5, 1, and 2 kHz] ≥ 15 dB) was 12.8%. In addition, the prevalence of any-HFHL and any-SFHL was 7.9% and 8.3%, respectively; that of unilateral HL, HFHL, and SFHL was 8.7%, 5.8%, and 5.9%, respectively; and that of bilateral HL, HFHL, and SFHL was 4.1%, 2.1%, and 2.4%, respectively. Detailed data are presented in Table 2.

**Table 2:** Prevalence of HL in Korean adolescents (n = 2,791).

	Number (Prevalence, %) [95% CI]	
Any HL	357 (12.8)	[11.57–14.09]
Any HFHL	220 (7.9)	[6.91–8.94]
Any SFHL	232 (8.3)	[7.31–9.40]
Unilateral HL	242 (8.7)	[7.65–9.78]
Unilateral HFHL	161 (5.8)	[4.93–6.70]
Unilateral SFHL	166 (5.9)	[5.10–6.89]
Bilateral HL	115 (4.1)	[3.41–4.93]
Bilateral HFHL	59 (2.1)	[1.61–2.72]
Bilateral SFHL	66 (2.4)	[1.83–3.00]

The PTA in the most affected ear is used to define any HL, and the PTA in the less affected ear is used to define bilateral HL. Any HL is considered present if bilateral HL is evident at either high or speech frequencies.

Participants exhibiting HL either at high frequencies (3, 4, 6, and 8 kHz) or speech frequencies (0.5, 1, and 2 kHz) are classified into these categories.

HL, hearing loss; CI, confidence interval; PTA, pure tone average; HFHL, high-frequency hearing loss; SFHL, speech-frequency hearing loss.

**Figure 2:**
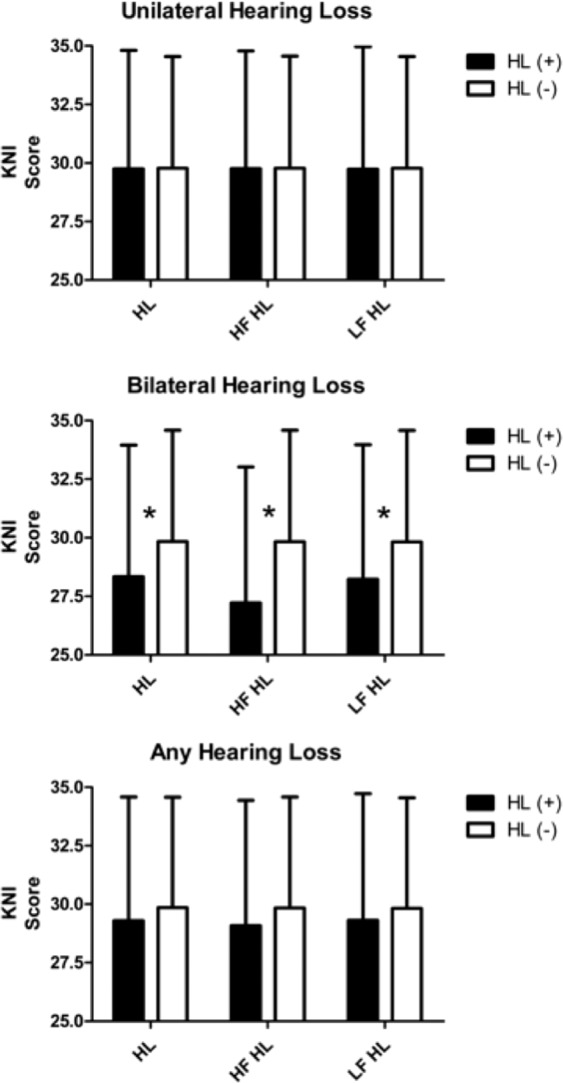
Analysis of the KNISE-Auditory Behavioral Checklist survey results. No significant difference was observed between students with and without hearing loss in the any hearing loss and unilateral hearing loss group. However, students with bilateral hearing loss, irrespective of whether they had high-frequency hearing loss or speech-frequency hearing loss, showed significantly worse performance scores than those without hearing loss. Moreover, students with bilateral high-frequency hearing loss showed relatively larger differences than those with other bilateral hearing loss.

**Figure 3:**
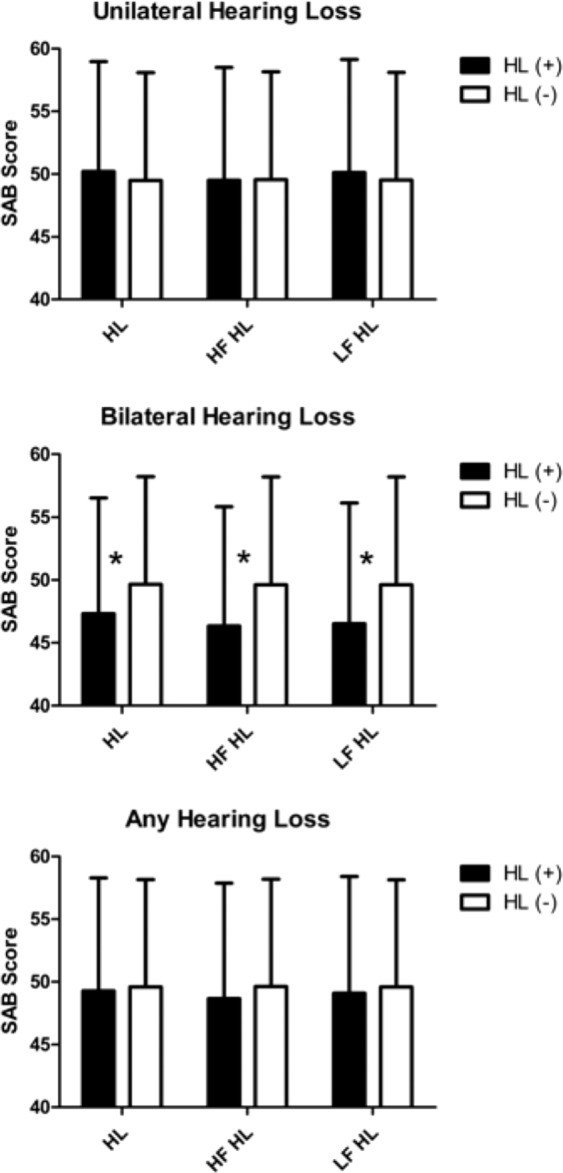
Analysis of the Scale of Auditory Behaviours survey results. No significant difference was observed between students with and without hearing loss in the any hearing loss and unilateral hearing loss groups. However, students with bilateral hearing loss, irrespective of whether they had high-frequency hearing loss or speech-frequency hearing loss, showed significantly worse performance scores than those without hearing loss. Moreover, students with bilateral high-frequency hearing loss showed relatively larger differences than those with other bilateral hearing loss.

**Figure 4:**
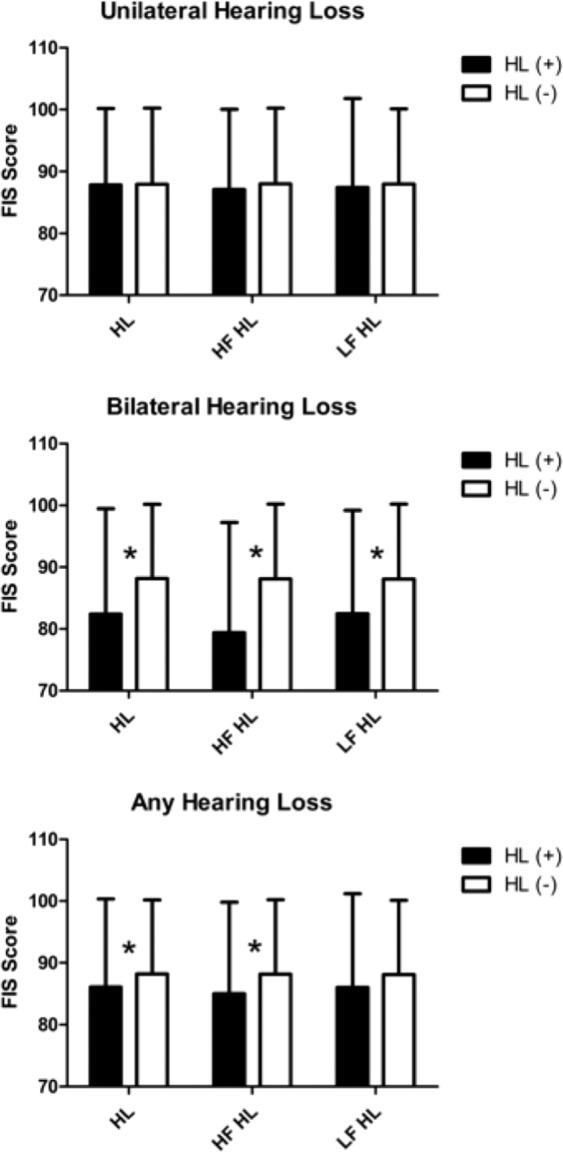
Analysis of Fisher’s Auditory Problems Checklist results. No significant difference was observed between students with and without hearing loss in the any hearing loss and unilateral hearing loss groups. However, students with bilateral hearing loss, irrespective of whether they had high-frequency hearing loss or speech-frequency hearing loss, showed significantly worse performance scores than those without hearing loss. Moreover, students with bilateral high-frequency hearing loss showed relatively larger differences than those with other bilateral hearing loss.

The KNISE-ABC analysis revealed no significant differences between students with and without hearing loss in the any-HL and unilateral HL groups. However, students with bilateral HL, irrespective of whether they had HFHL or SFHL, showed significantly worse performance scores than those without HL. Moreover, students with bilateral HFHL showed relatively larger differences than those with other bilateral HL (Fig. 2). The SAB analysis revealed no significant difference was observed between students with and without hearing loss in the any hearing loss and unilateral hearing loss groups. However, students with bilateral hearing loss, irrespective of whether they had high-frequency hearing loss or speech-frequency hearing loss, showed significantly worse performance scores than those without hearing loss. Moreover, students with bilateral high-frequency hearing loss showed relatively larger differences than those with other bilateral hearing loss **(**Fig. 3**)**. FAPC analysis also revealed no significant difference was observed between students with and without hearing loss in the any hearing loss and unilateral hearing loss groups. However, students with bilateral hearing loss, irrespective of whether they had high-frequency hearing loss or speech-frequency hearing loss, showed significantly worse performance scores than those without hearing loss. Moreover, students with bilateral high-frequency hearing loss showed relatively larger differences than those with other bilateral hearing loss **(**Fig. 4**)**.

## Discussion

In this study, the prevalence of SFHL and HFHL in the poorer ear was 11.6% and 10.3%, respectively, among Korean adolescents. There were no significant differences between the no-HL and any-HL groups in sex ratio, grade level of middle- vs. high school, household income, and family history, meaning that environmental factors such as noise exposure might be more influential on their hearing. In addition, when the three degrees of HL (any, bilateral, and unilateral) and mean scores from KNISE-ABC, SAB, and FAPC were compared, Korean adolescents with bilateral HL showed significantly worse scores on all three screening tests for APD.

Auditory behaviour is defined as behavioural characteristics associated with hearing that appear in relation to the process or outcome of receiving and perceiving auditory information in the peripheral and central auditory systems. Children’s hearing behaviour may vary according to the mechanism and function of the central nervous system (CNS). The ability to perceive and manipulate auditory information in the CNS can be used for sound localisation and lateralisation, auditory discrimination, determining the temporal aspects of audition (temporal resolution, temporal masking, temporal integration, and temporal ordering), assessing auditory performance with competing sound signals and acoustic signals, and assessing auditory performance with degraded acoustic signals^27^. One or more deficiencies in this ability are referred to as central APD or APD.

Auditory behavioural characteristics resulting from these central hearing processing problems can be caused by or related to difficulties in higher language, learning, and cognitive function. In addition, they may be associated with language disorders, reading disabilities, learning disabilities, and attention deficit hyperactivity disorder^27^. Children with hearing impairment may have problems with overall auditory behavioural characteristics and may exhibit sluggishness or difficulty in certain areas only.

However, distinguishing between APD and peripheral HL is sometimes difficult; moreover, both problems may sometimes coexist. Peripheral HL does not have uniform effects on the results of APD tests. Studies have shown that cochlear lesions can be clearly distinguished from cerebral lesions using the dichotic-digit test, dichotic sentence identification test, and frequency-pattern test^9,17^, whereas a less clear distinction can be made using low-pass filtered speech^17^, binaural interaction, and localisation/lateralisation^27^. The usefulness of the dichotic test of binaural integration in children was also doubted^28^. Previous studies have reported these relationships between APD and hearing impairment. Miltenberger *et al*.^29^ reported that auditory processing tests must always be related to the basic audiological assessment. They insisted that if the test results do not correspond with expectations based on the peripheral audiological assessment, the presence of APDs is likely, in addition to peripheral auditory disorders. Moreover, in the case of symmetrical hearing thresholds, auditory processing test results should be symmetrical. If the hearing thresholds are asymmetrical and performance in the better ear is poorer than that in the other ear, then an APD could be present^21^. The problem in APD is a decrease of speech performance in children with normal or mild hearing loss or functional hearing loss^12,30,31^.

In this study, adolescents with bilateral mild hearing loss showed a higher risk of APD. This may be because hearing loss is causally related to functional loss of central auditory processing^32^ or because the loss of the cortical auditory function and high frequency hearing loss occurred simultaneously for some reason. The latter case may be considered as having the same mechanism as loss of speech in noise processing and temporal processing from age-related hearing loss, leading to an increase in APD^33^. This idea requires further research.

In this study, we adopted three different survey tools—the KNISE-ABC, SAB, and FAPC—for evaluating the ability of central auditory processing among Korean adolescents. All three test results showed that students with bilateral HL, irrespective of whether they had HFHL or SFHL, had significantly worse performance scores than those without HL. Some authors have concluded that HL has only a slight influence on the dichotic-digit and pattern-recognition tests^34,35^, while Neijenhuis *et al*.^16^ reported that hearing-impaired patients did not show normal scores on these two tests. As with the results of Neijenhuis *et al*., in addition to the fact that peripheral sensorineural deafness affects the central hearing process, our results for bilateral HL are the most meaningful. We suggest that adolescents with bilateral sensorineural HL are more likely to have learning difficulties than those with normal or unilateral HL; therefore, more active hearing rehabilitation should be considered for such adolescents.

A limitation of this study is that the survey tests conducted to measure the capacity of the central auditory system were originally developed for evaluating children in the elementary school age group. The results of this study showed that only bilateral HL is related to disabilities in central auditory processing and learning; however, in reality, unilateral or various hearing impairments, such as SFHL or HFHL, may be related to learning disabilities in middle- and high-school students. These findings suggest the need for developing a survey to test central auditory processing in students of middle and high school age.

## Conclusions

Our findings suggest that hearing loss, especially bilateral high-frequency hearing loss, may affect central auditory processing. We should consider providing hearing aid, assistive listening devices, or enforcing educational programs to prevent further noise-induced hearing loss to improve the academic achievement of youth with bilateral hearing loss.

## Data Availability

All relevant data are within the manuscript and its supporting information files.
